# Changes in Oxidative Damage, Inflammation and [NAD(H)] with Age in Cerebrospinal Fluid

**DOI:** 10.1371/journal.pone.0085335

**Published:** 2014-01-14

**Authors:** Jade Guest, Ross Grant, Trevor A. Mori, Kevin D. Croft

**Affiliations:** 1 Australasian Research Institute, Sydney Adventist Hospital, Sydney, New South Wales, Australia; 2 Department of Pharmacology, School of Medical Sciences, Faculty of Medicine, University of New South Wales, Sydney, New South Wales, Australia; 3 Sydney Medical School, University of Sydney, Sydney, New South Wales, Australia; 4 School of Medicine and Pharmacology, Royal Perth Hospital Unit, University of Western Australia, Perth, Western Australia, Australia; University of Leipzig, Germany

## Abstract

An extensive body of evidence indicates that oxidative stress and inflammation play a central role in the degenerative changes of systemic tissues in aging. However a comparatively limited amount of data is available to verify whether these processes also contribute to normal aging within the brain. High levels of oxidative damage results in key cellular changes including a reduction in available nicotinamide adenine dinucleotide (NAD^+^), an essential molecule required for a number of vital cellular processes including DNA repair, immune signaling and epigenetic processing. In this study we quantified changes in [NAD(H)] and markers of inflammation and oxidative damage (F2-isoprostanes, 8-OHdG, total antioxidant capacity) in the cerebrospinal fluid (CSF) of healthy humans across a wide age range (24–91 years). CSF was collected from consenting patients who required a spinal tap for the administration of anesthetic. CSF of participants aged >45 years was found to contain increased levels of lipid peroxidation (F2-isoprostanes) (p = 0.04) and inflammation (IL-6) (p = 0.00) and decreased levels of both total antioxidant capacity (p = 0.00) and NAD(H) (p = 0.05), compared to their younger counterparts. A positive association was also observed between plasma [NAD(H)] and CSF NAD(H) levels (p = 0.03). Further analysis of the data identified a relationship between alcohol intake and CSF [NAD(H)] and markers of inflammation. The CSF of participants who consumed >1 standard drink of alcohol per day contained lower levels of NAD(H) compared to those who consumed no alcohol (p<0.05). An increase in CSF IL-6 was observed in participants who reported drinking >0–1 (p<0.05) and >1 (p<0.05) standard alcoholic drinks per day compared to those who did not drink alcohol. Taken together these data suggest a progressive age associated increase in oxidative damage, inflammation and reduced [NAD(H)] in the brain which may be exacerbated by alcohol intake.

## Introduction

Aging is an unavoidable biological process characterized by a progressive decline in physiological and biochemical function resulting in an increased predisposition to disease. In 1956 Harman proposed the oxidative stress theory of aging suggesting that the accumulation of unrepaired oxidative damage results in the typical aging phenotype [1]. The term ‘oxidative stress’ describes a significant imbalance between antioxidant defenses and the bodies’ formation of reactive nitrogen and/or oxygen species (ROS). While there are several sources of ROS within the body, the primary source is generally agreed to be the leakage of electrons to ground state oxygen from early components of the mitochondrial electron transport chain, resulting in the production of the superoxide radical (O_2_^•–^) [2], [3]. Importantly, at modest concentrations, ROS are used in a variety of normal physiological functions. Although there is the potential for damage, this is kept in check by an intricately connected antioxidant defense and repair system [4]. However, under conditions of reduced antioxidant capacity or excess production, ROS can cause indiscriminant damage to cellular constituents (DNA, proteins and lipids) that, if unrepaired, may lead to cell death and tissue dysfunction.

The brain is particularly vulnerable to oxidative damage as a consequence of its high oxygen demand, high level of both polyunsaturated fatty acids and transition metals, and poor antioxidant defenses [5]–[7]. As we age, the vulnerability of the brain to oxidative damage increases due to reduced integrity of the blood brain barrier and amplified mitochondrial dysfunction [8], [9]. Indeed animal and tissue studies have shown the aging brain to be accompanied by an accumulation of markers of lipid, protein and DNA oxidative damage [10]–[12]. Failure to repair this damage has been demonstrated to cause genomic instability and neuronal apoptosis and is associated with the development of neuropathologies such as Alzheimer’s disease, Parkinson’s disease, and amyotrophic lateral sclerosis [13]–[17].

Both normal brain aging and neurodegenerative disease are characterized by increased inflammation associated with microglial over activation and a subsequent rise in pro-inflammatory cytokines [18]–[21]. Excessive release of pro-inflammatory cytokines further promotes a pro-oxidative state and neuronal degradation. Elevated levels of the inflammatory cytokine IL-6 have been associated with cognitive impairment and the induction of Alzheimer’s-type hyperphosphorylation of tau protein [22], [23].

As inflammation and oxidative damage rise with age a decrease in available nicotinamide adenine dinucleotide (NAD^+^) has been observed in multiple organs of the rat [24], including the brain (data unpublished). NAD^+^ is a ubiquitous molecule that is required for a number of vital cellular processes. In addition to its role in cellular energy and metabolism there are several enzymes, including poly(ADP-ribose) polymerase 1 (PARP) and silent information regulators (e.g. SIRT1), that use NAD^+^ as their substrate [25]–[27]. Importantly PARP activation in response to DNA damage catalyzes the successive cleavage of the ADP-ribose moiety from NAD^+^ resulting in the formation of poly(ADP-ribose) subunits. Under conditions of mild-to-moderate DNA damage this process facilitates DNA repair [28]. However over-activation of PARP, due to excessive DNA damage, can result in neuronal death as a consequence of decreased ATP production due to NAD^+^ depletion [29]–[31]. In order to preserve cellular energy and concomitantly SIRT1 (associated with maintaining cellular longevity) and PARP activity, adequate levels of NAD^+^ must be sustained.

Inflammation and oxidative activity, when adequately regulated, form part of the normal physiology in all age groups. However during the course of life, each individual will experience (to varying degrees) a gradual increase in oxidative damage burden in many of the body’s tissues. If experienced in the brain, neurodegenerative changes are likely. While age is the major risk factor for the development of most neurodegenerative disorders [32], [33], a number of lifestyle choices can also promote pathogenesis by increasing oxidative stress. Previous studies have indicated that chronic alcohol exposure in humans’ results in neurodegeneration, ranging from minor synaptic and dendritic changes to neuronal cell death [34]. More recently administration of alcohol to rats was shown to significantly increase brain mitochondrial lipid and protein oxidation and decrease superoxide dismutase mRNA expression and ATP-ase activity. The authors postulated that the alcohol-induced production of ROS alters mitochondrial membrane properties leading to mitochondrial dysfunction and subsequently further ROS production [35].

Collectively these reports indicate that certain lifestyle choices may accelerate the development of an age associated oxidative-inflammatory state leading to increased tissue damage and reduced DNA repair capacity (through reduced NAD^+^ availability) within the central nervous system. While evidence from cell culture, animal and limited postmortem brain tissue studies support this hypothesis, to date no study has investigated this in a healthy human cohort.

In this study we investigated whether markers of oxidative and inflammatory activity increase with age in the central nervous system of relatively healthy humans and whether this was associated with a decrease in available NAD(H). We further correlated changes in CSF oxidative/inflammatory markers and [NAD(H)] with specific modifiable lifestyle factors.

## Materials and Methods

### Ethics Statement

This study was conducted in accordance with the Helsinki declaration. Ethical approval was obtained from the Human Research Ethics Committee, Sydney Adventist Hospital (HREC# 2011-005). Written informed consent was obtained from all participants prior to commencement.

### Participants

Male (n = 20) and female (n = 50) participants, who required a spinal tap for the administration of anesthetic as part of routine care, were recruited at Sydney Adventist Hospital, Australia. The average age of participants was 53 years (SD = 19.9, interquartile range = 38). Participants were excluded from the cohort if they were smokers or had a confirmed diagnosis of a neurological/neurodegenerative disorder or central nervous system infection. In total 70 CSF and 38 matched blood samples were collected from consenting participants considered in general good health.

### Sample Collection

Fasting (≥10 h) blood and CSF samples were collected by an accredited anesthetist no longer than 30 minutes apart. CSF samples were collected, prior to injection of spinal anesthetics, via standard lumber puncture. Blood samples were collected into heparinized tubes from an intravenous cannula inserted into a superficial vein on an upper limb, prior to the administration of fluids or anesthetics. Samples were prepared by centrifuging at 1800 rpm for 10 minutes and stored within 1 hour of collection, at −194 degrees Celsius until analysis. Samples intended for F2-isoprostane analysis where stored in the presence of a glutathione/butylated hydroxytoluene preservative.

### Assessment of Alcohol Consumption

Alcohol consumption was assessed via questionnaire upon hospital admission. Specifically participants were asked ‘*Do you drink alcohol.’* If this was affirmative participants were asked to provide the number of standard drinks consumed per day.

### Total NAD(H)

Total NAD(H) concentrations in plasma and CSF samples were measured spectrophotometrically using a thiazolyl blue microcycling assay established by Bernofsky and Swan (1973) [36], and adapted to a 96 well plate format by Grant and Kapoor (1998) [37]. In brief, 125 µL of the reaction mixture containing 120 mM bicine (pH 7.8), 0.5 mM MTT, 2 mM PMS, 0.6 M ethanol and alcohol dehydrogenase (300 units/mL) was added to either 6 µL of plasma or 20 µL of CSF. Following a 10 minute incubation at 37 degrees Celsius, the concentration of total NAD(H) was measured, using a Model 680XR microplate reader (BioRad, Hercules), as the change in absorbance at 570 nm.

### Total Antioxidant Capacity (TAC)

CSF total antioxidant capacity was measured using a standardized commercial kit (Antioxidant Assay Kit, Cayman Chemical Company, Ann Arbor, MI USA). This assay relies on the ability of antioxidants to inhibit oxidation of 2,2′-Azino-di-[3-ethylbenzthiazoline sulphonate] (ABTS) by metmyoglobin. Briefly, 10 µL of metmyoglobin was added to 10 µL of diluted sample. 150 µL of chromagen, containing ABTS, was subsequently added and the reaction initiated by adding 40 µL of 441 µM hydrogen peroxide. The plate was incubated on a shaker for 5 minutes and the amount of oxidized ABTS was measured spectrophotometrically, at an absorbance of 750 nm, using a Model 680XR microplate reader (BioRad, Hercules).

### Interleukin-6 (IL-6)

IL-6 was measured using a standardized commercial solid phase sandwich enzyme linked-immuno-sorbent assay (ELISA) (Human IL6 High Sensitivity ELISA Kit, Abcam, Cambridge, MA USA). Briefly, 100 µL of CSF was added to a plate pre-coated with a monoclonal antibody specific for IL-6. 50 µL of biotinylated anti-IL6 was then added and the plate incubated for 3 hours. After incubation, the plate was washed before the addition of the enzyme, horseradish peroxidase. The plate was incubated for 30 minutes and then washed again to remove any unbound enzymes. The 3,3′,5,5′-Tetramethylbenzidine substrate was added and the plate was incubated in the dark for 12–15 minutes, after which H_2_SO_4_ was added to the wells to stop the enzyme-substrate reaction. The intensity of this colored product is directly proportional to IL-6 concentration. Absorbance was measured at 450 nm using a Model 680XR microplate reader (BioRad, Hercules).

### 8-hydroxy-2′-deoxyguanosine (8-OHdG)

8-OHdG was measured in CSF samples using a standardized commercial competitive ELISA (Highly Sensitive 8-OHdG Check, Japan Institute for the Control of Aging, Shizuoka Japan). Briefly 50 µL of sample or standard and 50 µL monoclonal antibody was adsorbed onto a 96-well plate precoated with 8 OHdG. Following an overnight incubation at 4 degrees Celsius the plate was washed and incubated with a secondary antibody for 1 hour. The plate was washed again before the addition of a chromatic solution for 15 minutes, after which the reaction was terminated and absorbance was measured at 450 nm using a Model 680XR microplate reader (BioRad, Hercules).

### F2-Isoprostanes

Total F2-Isoprostanes were measured in CSF by gas chromatography–mass spectrometry (GC-MS) using electron capture negative ionization according to a modification in the method of Mori et al (1999) [38]. Briefly, after the addition of an internal standard (15-F2t-IsoP-d4, 5 ng), plasma and CSF samples (200 µL) were hydrolyzed with KOH in methanol, acidified, and applied to prewashed Certify II cartridges (Varian). Following washing with methanol:water (1∶1) and hexane:ethyl acetate (75∶25) the F2-Isoprostanes were eluted with ethyl acetate:methanol (90∶10), dried, and derivatized. The F2-Isoprostanes were quantitated by monitoring ions at m/z 569 and 573 for F2-Isoprostanes and 15-F2t-IsoP-d4 respectively.

### Statistical Analysis

Statistical analyses were performed using SPSS version 16.0 and GraphPad Prism version 5 for Windows. Data is presented as means ± standard deviation unless otherwise stated. Multiple linear regression, controlling for age, was used to identify significant relationships between CSF [NAD(H)], plasma [NAD(H)], and markers of oxidative damage and inflammation. The Independent T Test or Mann-Whitney U Test was employed to analyze the effect of age on markers of oxidative damage, inflammation and metabolism. The Wilcoxon Signed Ranks Test was used to identify the association between mean plasma and CSF NAD(H) levels. Kruskal-Wallis with Dunn’s post-hoc test was used to assess the effect of alcohol consumption on markers of oxidative damage, inflammation and metabolism. Both the Kolmogorov-Smirnov, Shapiro-Wilk and histogram analysis was used to check normality of the variables. When required the Levene’s Test of Equality was used to check homogeneity of variances between groups. If the variances of the groups were found to be either not homogenous and/or normality tests for the variables were not significant then further investigation with graphical displays was performed to assess the distributions of the variables. Both adjusted and non-adjusted P-values are provided throughout with test significance set at P value ≤0.05.

## Results

### Age Associated Differences in CSF Markers of Oxidative and Inflammation

A number of studies have shown that lifestyle behaviors in midlife (i.e. around 45–50 years) are associated with reduced cognitive function in later life [38]–[42]. Thus to assess age related differences in CSF markers of oxidative damage and inflammation, for this analysis participants were divided into two groups, aged ≤45 years and >45 years.

In this cohort older age was associated with an increase in a number of CSF oxidative and inflammatory markers (Table 1). Specifically CSF lipid peroxidation (F2-isoprostane) was significantly increased in those aged >45 years; 417.49±34.39 pmol/L compared to those ≤45 years; 395.09±34.04 pmol/L (p = 0.04). Those over 45 years also showed significantly increased levels of the inflammatory cytokine IL-6 (p = 0.00, 2.37±1.93 vs. 0.71±0.43 pg/mL for those ≤45 years) and reduced CSF TAC levels (p = 0.00, 0.90±0.28 vs. 1.49±0.51 nmol/mg protein for those <45 years). Those aged >45 years also tended to have raised levels of CSF 8-OHdG, a marker of oxidative DNA damage, although this did not quite reach statistical significance (p = 0.06) (Table 1).

**Table 1:** Differences in selected CSF markers according to age and gender.

	≤45 years, mean (± SD)			>45 years, mean (± SD)		
Characteristic/Test	Male+Female	Male	Female	Male+Female	Male	Female
**(n)**	34	1	33	36	19	17
**Average Age (years)**	34 (5)	45	34 (5)	71 (8)	70 (8)	73 (9)
**CSF F2-Isoprostanes (pmol/L)**	395.09(34.04)	405.95	394.57 (34.79)	417.49 (34.39)*	422.08 (39.45)	413.23 (29.81)
**CSF 8-OHdG (ng/mL)**	0.45 (0.09)	.	0.45 (0.09)	0.52 (0.13)	0.53 (0.16)	0.51 (0.12)
**CSF IL-6 (pg/mL)**	0.71 (0.43)	1.25	0.69 (0.43)	2.37 (1.93)**	2.91 (2.26)	1.71 (1.23)**
**CSF TAC (nmol/mg protein)**	1.49 (0.51)	.	1.49 (0.51)	0.90 (0.28)**	0.87 (0.30)	0.98 (0.30)*
**CSF [NAD(H)] (µg/mL)**	88.59 (21.07)	42.68	89.98 (19.75)	75.88 (30.14)*	78.64 (34.38)	73.13 (25.99)*
**Plasma [NAD(H)] (µg/mL)**	374.93 (102.94)	471.18	369.26 (103.18)	345.01 (94.95)	362.02 (38.26)	332.26 (79.98)

Due to small sample volume, some tests have one or more missing data. Comparisons made using the Independent T Test or Mann-Whitney U Test.

p≤0.05 compared to ≤45 years,

p≤0.001 compared to ≤45 years.

Assessing these trends in each gender revealed that the CSF of females >45 years contained significantly higher levels of IL-6 (p = 0.00, 1.71±1.23 vs. 0.69±0.43 pg/mL) and lower TAC levels (p = 0.00, 0.98±0.30 vs. 1.49±0.51 nmol/mg protein) than their younger counterparts. Due to low number of male participants ≤45 years, valid comparisons were not possible.

### Age Associated Decrease in CSF NAD(H)

CSF NAD(H) levels were significantly lower in participants aged >45 years compared to those aged ≤45 years; 75.88±30.14 vs. 88.59±21.07 µg/mL respectively (p = 0.05) (Table 1). Assessing these trends in each gender revealed that, the CSF of females >45 years contained significantly lower levels of NAD(H) compared to their younger counterparts (p = 0.01, 73.13±25.99 vs. 89.98±19.75 µg/mL respectively). Due to low number of male participants ≤45 years, valid comparisons were not possible.

### Inter-correlation between CSF NAD(H), Oxidative Damage and Inflammatory Markers

**Figure 1:**
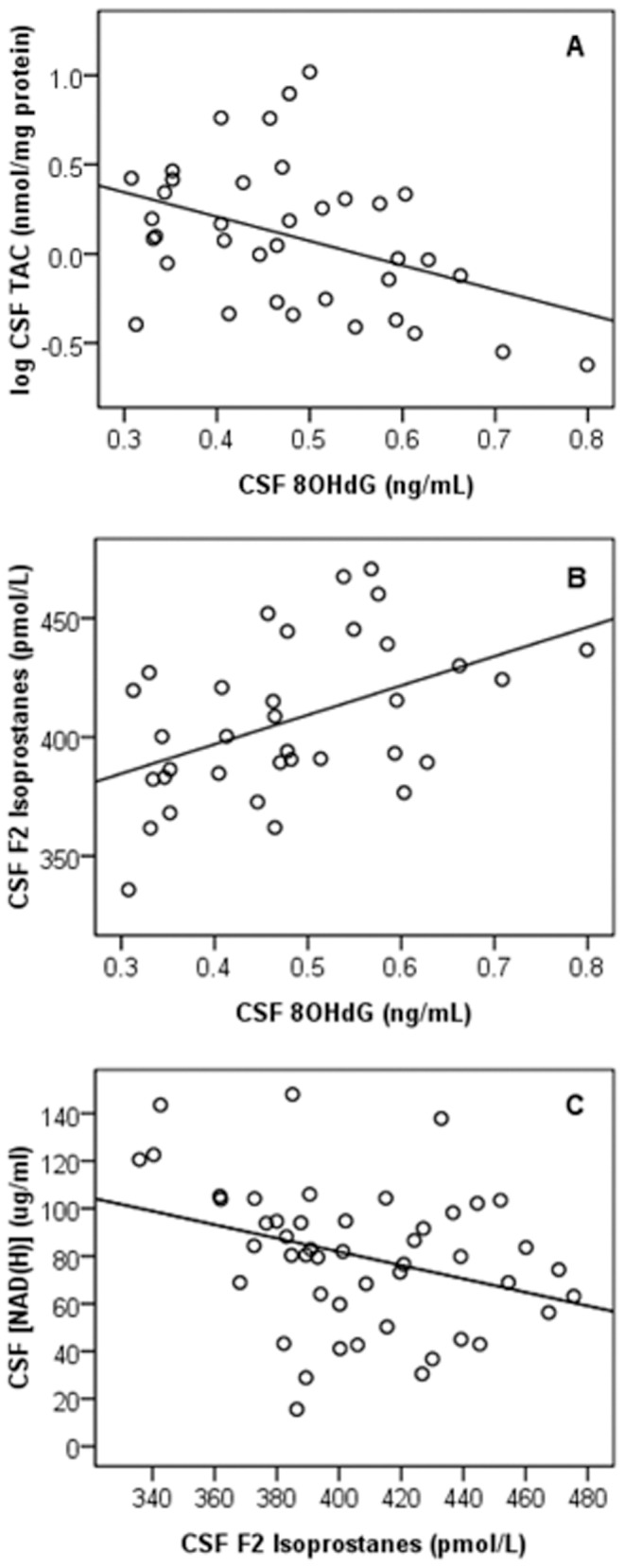
Inter-correlation between CSF NAD(H), oxidative damage and inflammatory markers. (A) Positive association between CSF 8 OHdG and CSF total antioxidant capacity. A significant positive association was observed between CSF 8(TAC) (p = 0.05, n = 37). Comparisons were made using multiple linear regression controlling for age. **(B) Positive association between CSF 8 OHdG and CSF F2 Isoprostane levels.** A significant positive association was observed between CSF 8 OHdG and F2 Isoprostane levels (p = 0.01, n = 34). Comparisons were made using multiple linear regression controlling for age. **(C) Inverse association between CSF [NAD(H)] and CSF F2 Isoprostane levels.** A significant inverse association was observed between CSF [NAD(H)] and F2 Isoprostane levels (p = 0.02, n = 48). Comparisons were made using the Pearson correlation coefficient and multiple linear regression controlling for age.

After controlling for age, a significant inverse association was observed between CSF log TAC and CSF 8 OHdG (p = 0.05, n = 37) (Figure 1A). A significant positive association was observed between CSF DNA (8 OHdG) and lipid oxidation (F2-isoprostane) markers (p = 0.01, n = 34) (Figure 1B). An inverse association between CSF [NAD(H)] and F2-isoprostane levels was also found (p = 0.02, n = 48) although this did not remain statistically significant after controlling for age (p = 0.06) (Figure 1C). No further associations were apparent between CSF [NAD(H)], oxidative damage and inflammatory markers.

### CSF [NAD(H)] Correlates with Peripheral [NAD(H)]

**Figure 2:**
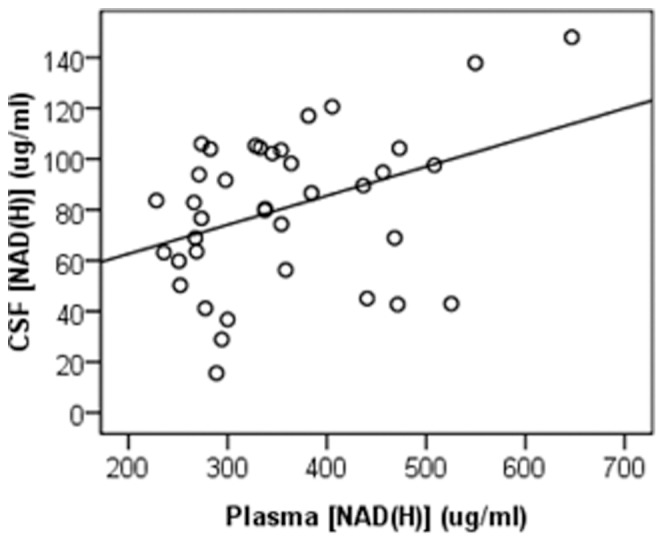
Positive association between plasma and CSF NAD(H) levels. A significant positive relationship was observed between plasma and CSF NAD(H) concentrations (p = 0.03, n = 38). An increase of one µg/mL in plasma [NAD(H)] was associated with a 0.11 µg/mL increase in CSF [NAD(H)]. Comparisons were made using multiple linear regression controlling for age.

After controlling for age, a significant positive relationship was observed between plasma and CSF NAD(H) concentrations (p = 0.03, n = 38) (Figure 2). An increase of one µg/mL in plasma [NAD(H)] was associated with a 0.11 µg/mL increase in CSF [NAD(H)]. The mean level of NAD(H) was significantly lower in CSF (82.24±26.59 µg/mL, n = 38) compared to plasma (358.81±98.56 µg/mL, n = 38) (p = 0.00).

### Influence of Alcohol Intake on CSF [NAD(H)], Oxidative Damage and Inflammation

**Figure 3:**
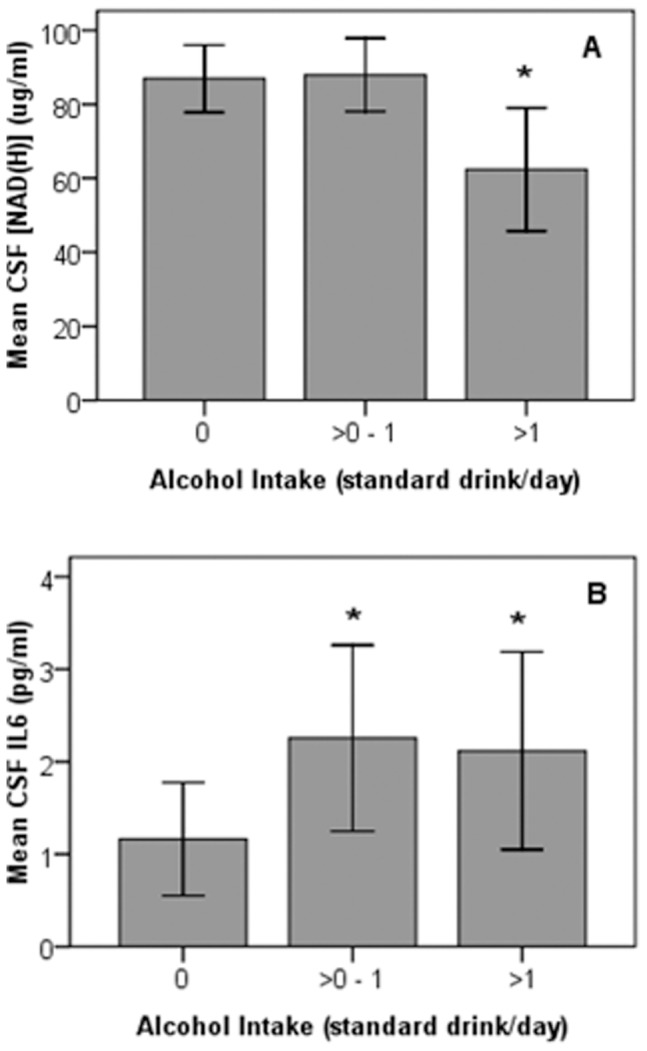
Association between alcohol consumption and CSF (A) [NAD(H)] (B) IL-6 levels. (A) Alcohol consumption is associated with decreased levels of CSF NAD(H). Participants who consumed zero (n = 32), >0–1 (n = 14) and >1 (n = 8) standard alcoholic drinks per day were found to contain significantly different levels of CSF NAD(H) (p = 0.02). Specifically the CSF of participants who consumed >1 standard drink of alcohol per day contained significantly lower levels of NAD(H) compared to those who reported consuming zero drinks per day; 62.39±19.93 vs. 86.93±25.32 µg/mL respectively (p<0.05). Comparisons were made using the Kruskal-Wallis with Dunn’s post-hoc test. Error bars represent 95% confidence intervals. **(B) Alcohol consumption is associated with increased levels of CSF IL-6.** A significant increase in CSF IL-6 was observed in participants who drank >1 (p<0.05) and >0–1 (p<0.05) standard alcoholic drinks per day compared to those who drank zero; 2.12±1.28, 2.25±1.66 vs. 1.16±1.67 pg/mL respectively. Comparisons were made using the Kruskal-Wallis with Dunn’s post-hoc test. Error bars represent 95% confidence intervals.

The data was further analyzed to identify possible relationships between alcohol intake and CSF [NAD(H)] and markers of inflammation and oxidative damage. For this analysis, participants were divided into three groups, those who consumed zero (n = 32), >0–1 (n = 14) and >1 (n = 8) standard alcoholic drinks per day. CSF NAD(H) levels were significantly different between the groups (p = 0.02). Specifically the CSF of participants who consumed >1 standard drink of alcohol per day contained significantly lower levels of NAD(H) compared to those who reported consuming zero drinks per day; 62.39±19.93 vs. 86.93±25.32 µg/mL respectively (p<0.05) (Figure 3A). A significant increase in CSF IL-6 was also observed in participants who drank >1 (p<0.05) and 0–1 (p<0.05) standard alcoholic drinks per day compared to those who did not consume alcohol; 2.11±1.28, 2.25±1.66 vs. 1.16±1.67 pg/mL respectively (Figure 3B). No associations were found between alcohol consumption and CSF markers of DNA (8-OHdG) and lipid (F2-isoprostanes) oxidative damage or total antioxidant capacity. When the data was stratified according to both age and gender CSF IL6 levels remained significantly higher in female participants who drank >0–1 or ≥1 standard alcoholic drinks per day. No other observations remained statistically significant after stratifying for age and gender.

### Gender Associated Differences in CSF Markers of Oxidative Damage and Inflammation

In this cohort, the CSF of male participants (n = 18) contained significantly higher levels of IL-6 compared to females (n = 42) (p = 0.00); 2.82±2.23 vs. 1.03±0.91 pg/mL respectively. However when the data was stratified into age groups, this observation did not remain statistically significant (Table 1). No significant differences were observed between males and females for any other markers of inflammation, oxidative damage or [NAD(H)].

## Discussion

Considerable evidence now indicates that both inflammation and oxidative stress contribute to the development of various neuropathologies including Alzheimer’s and Parkinson’s disease [14], [16], [23]. While age is the major risk factor for the development of most neurodegenerative disorders it has yet to be confirmed if oxidative stress and inflammation increase during normal brain aging in humans. To our knowledge this is the first study to show that both oxidative damage and inflammation increase after the age of 45 in the central nervous system (CNS) of relatively healthy humans. In this study we report that the CSF of participants aged over 45 years contained statistically higher amounts of the oxidative damage marker F2-isoprostane and the inflammatory cytokine IL-6. Those aged over 45 years also tended to have increased CSF levels of the DNA damage marker 8-OHdG. These data are consistent with previous results from both our laboratory and others showing that DNA and lipid oxidation increase with age in multiple organs, including the brain in animals [11], [12], [24], [43]. While limited research has been conducted within the CNS of living humans, an age related accumulation in markers of both oxidative damage (8-OHdG) and inflammation (IL-6) has been previously reported in postmortem brain tissue [9], [44].

It is well established, that oxidative DNA damage activates the NAD-dependent DNA repair enzyme, PARP, which is involved in base excision repair [45]. Utilizing unexposed human skin, our laboratory has previously shown that that PARP activity increases with age and correlates with NAD^+^ depletion [46]. In the present study we investigated whether levels of CSF NAD(H) were also associated with age and report for the first time that [NAD(H)] does decline with age in the CNS of healthy humans. As expected an inverse trend between CSF [NAD(H)] and markers of central DNA (8-OHdG) and lipid (F2-isoprostanes) oxidative damage was also observed. In addition, as would be predicted, after controlling for age, increased CSF total antioxidant capacity was significantly correlated with higher CSF levels of NAD(H).

Adequate levels of NAD(H) are required to maintain normal cellular functions. In addition to its role in cellular energy metabolism there are several enzymes, in addition to PARP, that use the oxidized form, NAD^+^ as the sole substrate for their activities [25]–[27]. Notably SIRT1, a member of a highly conserved family of histone deacetylases modulates key transcription factors such as FOXO and pro-apoptotic p53, and is thought to play a central role in cell longevity and aging [47], [48]. As both PARP and SIRT1 compete for the same intracellular pool of NAD^+^ it has been suggested that depletion of NAD^+^ results in reduced SIRT1 deacetylase activity [24], [49]. Further PARP over-activation has been shown to reduce ATP production due to NAD^+^ depletion resulting in neuronal death [29]–[31]. The theory that excessive NAD^+^ depletion facilitates cell death is supported by observations in rodent models of brain ischemia and Alzheimer’s disease where significantly reduced levels of total cellular NAD^+^ occur prior to neuronal death [50]–[52]. Adequate NAD^+^ levels are therefore required to maintain cellular energy and robust SIRT1 activity. However further functional studies are required to determine the level of biochemical impact the relatively modest (∼14%) decrease in NAD(H) levels would have on cell metabolism.

While the physiologic and pathologic importance of NAD^+^/NADH dependant mechanisms’ in both the central nervous system and periphery is apparent, it was not previously known whether peripheral stores of NAD(H) influence central NAD(H) levels. This study is the first to show a positive correlation between matched CSF and plasma NAD(H) levels in a healthy human cohort. This is consistent with a previous study by Rex and colleagues (2002) who observed that NAD(H) in both its oxidized and reduced forms is capable of crossing the blood brain barrier in rats [53]. While evidence indicates that the brain is capable of independently synthesizing NAD^+^ [54], [55], results from the present study suggest that NAD(H) levels in the brain are potentially influenced by peripheral NAD(H) levels and consequently lifestyle choices that affect the peripheral NAD(H) pool.

Although age is the major risk factor for the development of most neurodegenerative disorders [32], [33], a number of lifestyle choices can increase central oxidative damage and inflammation and thereby promote disease. In this cohort we observed that consumption of less than half a glass or more of alcohol per day was associated with a statistically significant increase in CSF levels of the inflammatory cytokine IL-6. While excessive alcohol consumption is generally agreed to cause alteration in brain structure, function and loss of brain mass [56], [57], the effect of low to moderate alcohol consumption on brain health is still debated within the literature. Some authors suggest that low to moderate alcohol consumption may improve cognitive functioning and even reduce the risk of Alzheimer’s disease [58], [59]. In contrast to these largely epidemiological studies, a meta-analysis conducted by Verbaten (2009) assessing the effects of alcohol consumption on brain integrity, determined by both MRI and cognitive performance, concluded that the consumption of even light to moderate doses of alcohol lead to shrinkage of the brain and to decreases in grey matter volume [60].

The suggested pathways by which alcohol may damage the brain are numerous and include, but are not limited to, disruption of neural cell adhesion molecules [61]–[63], promotion of endoplasmic reticulum protein misfolding [64], neuronal hypersensitivity to excitotoxic insults [65], reduction of endogenous antioxidants [66], and increased free radical damage to both blood brain barrier endothelial cells as well as neurons [67], [68]. Consistent with our findings, others have also shown that alcohol, even at low/moderate concentrations, can act as a ligand for toll like receptor 4 (TLR4), stimulating the mitogen-activated protein kinases (MAPKs) and the transcription factor NF-κB pathways, leading to the production of nitric oxide and inflammatory cytokines [69], [70].

We also report for the first time an inverse relationship between alcohol consumption and CSF [NAD(H)]. Specifically the CSF of participants who consumed greater than one glass of alcohol per day had significantly lower levels of NAD(H) compared to those who did not drink alcohol. While research investigating the effect of alcohol on NAD(H) is scarce our results are consistent with a very early report by McElfresh and McDonald (1983) who also observed in *Drosophila* that NAD^+^ levels decrease by at least 20% in response to ethanol stress [71]. Additionally recent data from our laboratory (unpublished) indicates that acute ethanol exposure (10 mM, equivalent to a blood alcohol of 0.05%) decreases intracellular [NAD(H)] in cultured human primary astrocytes (brain metabolic support cells) by as much as 64% within 30 minutes in conjunction with an increase in oxidative damage and PARP activity. By increasing CNS inflammation and oxidative damage, alcohol consumption may stimulate PARP over-activation and subsequently decrease central NAD(H) levels promoting senescence and neurodegeneration.

While the observations reported in this study are statistically valid it is recognized that these associations have been obtained from a modest number of participants (38 plasma/CSF matched and 70 CSF). Though both genders were well represented in the older age groups, due to the difficulty in obtaining CSF samples from essentially healthy individuals greater numbers of females were represented in the younger age range. The disproportionately low number of younger male participants, as well as the small number of participants for which alcohol consumption data was available, prevented a comprehensive analysis on how gender may influence our findings. Self-reported alcohol consumption was also relied upon in this study and may have introduced some error into our analysis. However while such error may cause the levels of alcohol reported to slightly differ from the number of glasses actually consumed, it is unlikely to significantly affect the rank order of participants. Finally the restricted volume of sample collected as part of this study limited our analytical profile negating comparisons with other important molecular species such as the range of anti-inflammatory cytokines. Future studies overcoming these limitations are required to verify the consistency of our observations.

## Conclusion

An extensive body of evidence indicates that oxidative stress and inflammation play a central role in the physiology of aging. However, comparatively limited data are available to verify whether these processes also contribute to normal aging within the brain. This study reports for the first time a potential link between aging, increased oxidative stress, inflammation and alcohol consumption and a decline in the essential pyridine nucleotide [NAD(H)], in the CSF of a healthy human population. We also provide evidence of a relationship between peripheral and central NAD(H) stores.

Taken together these data suggest a progressive age associated increase in oxidative damage, inflammation and reduced NAD(H) in the brain which may be exacerbated by certain lifestyle choices such as regular alcohol consumption.

As reduced NAD(H) levels impact at least PARP and SIRT1 activity the observed decrease in NAD(H) availability within the aging brain may facilitate cell metabolic and genomic instability increasing an individuals’ susceptibility to degenerative disease. However further follow-up of the participants characterized in this study is required to confirm this hypothesis.
